# NmTHC: a hybrid error correction method based on a generative neural machine translation model with transfer learning

**DOI:** 10.1186/s12864-024-10446-4

**Published:** 2024-06-07

**Authors:** Rongshu Wang, Jianhua Chen

**Affiliations:** Department of Electronic Engineering, Information School, Yunnan University, Kunming, Yunnan China

**Keywords:** Long read, Hybrid error correction, Neural machine translation, Natural language processing

## Abstract

**Backgrounds:**

The single-pass long reads generated by third-generation sequencing technology exhibit a higher error rate. However, the circular consensus sequencing (CCS) produces shorter reads. Thus, it is effective to manage the error rate of long reads algorithmically with the help of the homologous high-precision and low-cost short reads from the Next Generation Sequencing (NGS) technology.

**Methods:**

In this work, a hybrid error correction method (NmTHC) based on a generative neural machine translation model is proposed to automatically capture discrepancies within the aligned regions of long reads and short reads, as well as the contextual relationships within the long reads themselves for error correction. Akin to natural language sequences, the long read can be regarded as a special “genetic language” and be processed with the idea of generative neural networks. The algorithm builds a sequence-to-sequence(seq2seq) framework with Recurrent Neural Network** (**RNN) as the core layer. The before and post-corrected long reads are regarded as the sentences in the source and target language of translation, and the alignment information of long reads with short reads is used to create the special corpus for training. The well-trained model can be used to predict the corrected long read.

**Results:**

NmTHC outperforms the latest mainstream hybrid error correction methods on real-world datasets from two mainstream platforms, including PacBio and Nanopore. Our experimental evaluation results demonstrate that NmTHC can align more bases with the reference genome without any segmenting in the six benchmark datasets, proving that it enhances alignment identity without sacrificing any length advantages of long reads.

**Conclusion:**

Consequently, NmTHC reasonably adopts the generative Neural Machine Translation (NMT) model to transform hybrid error correction tasks into machine translation problems and provides a novel perspective for solving long-read error correction problems with the ideas of Natural Language Processing (NLP). More remarkably, the proposed methodology is sequencing-technology-independent and can produce more precise reads.

## Background

NGS technologies generate precise yet short reads, typically with a maximum length of around 600 bases, which poses significant challenges for subsequent reconstruction and analysis processes [1]. Third-generation sequencing (TGS) technologies, exemplified by PacBio and Nanopore, generate long reads spanning up to 10 ~ 15kbp which provides a chance to solve challenging downstream problems such as de novo assembly [2], variant calling [3]. The PacBio platform generates Continuous Long Reads(CLR), exhibiting ultra-length but a high error rate ~ 13% [4], and Circular Consensus Sequencing(CCS) reads with high accuracy but shorter length (e.g., median = 423 bp, max = 1,915 bp) [5]. Nanopore technology cannot sequence the same molecule multiple times as PacBio, and the error rates of Nanopore reads are ~ 15% [6]. Although the error rates of “single-pass” long reads from the two platforms are unsatisfactory, their exceptionally read lengths confer an irreplaceable advantage in downstream analysis. Algorithmically managing the error rates for the vast amount of accumulated “single-pass” sequencing data in recent years is both economical and imperative. Therefore, combining existing homologous high-precision short reads with carefully designed algorithms to correct long reads is an cost-effective way, which has successfully aroused the interest of many researchers [7].

As highlighted in the survey of long-read error correction [8], the integration of high-precision information from short reads with the global information of long reads contributes to enhancing the accuracy and robustness of long-read error correction. Existing hybrid error correction algorithms can be divided into four types: short-read alignment-based, short-read assembly based, De Bruijn graph (DBG)-based, and Hidden Markov Models (HMMs)-based. Short-read alignment-based methods involve aligning short reads to long reads and computing a consensus sequence to rectify the corresponding interval in the long read. PacBioToCA [9], Proovread [10], Nanocorr [11], ColorMap [12], HECIL [13], etc. are based on this strategy. However, aligning short reads to particularly repetitive and noisy regions of long reads is a challenging task. To address this issue, assembly-based methods pre-assemble short reads into longer contigs. ECTools [14], HALC [15], MiRCA [16] are based on this strategy, leveraging the contextual information from adjacent regions post-assembly to facilitate the effective alignment of contigs to repetitive and noisy regions in long reads. The DBG-based approaches leverage the DBG constructed from short-read k-mers to avoid the intricate assembly process. Subsequently, they anchor the long reads to the DBG and traverse the graph to obtain an optimal path. LoRDEC [17], Jabba [18], FMLRC [19], ParLECH [20] are based on this strategy.

Unfortunately, most of the algorithms mentioned above suffer from at least one of the following limitations: 1) Different algorithm is designed based on the distinct error profile of the reads from different platforms, leading to a discounted performance when applied to the data from another platform. 2) The majority of algorithms ignore those fragments that cannot be aligned with any short read, which can compromise the continuity and the length of long reads. 3) There is some human preference setting in traversing the optimal path in DBG based algorithms. Consequently, *Canlkan *et al. proposed the only completely data-driven machine learning-based hybrid correction algorithm, named Hercules [21]. It models each complete long read as a Hidden Markov Model (HMM), and refine the parameters automatically based on the error profiles of error-prone sequencing technologies. As the only machine learning-based approach, Hercules is adept at capturing short-term dependencies inherent in neighboring regions in a sequence. Nevertheless, HMMs exhibit limitations in capturing long-term dependencies due to their reliance on the assumption of a finite state space. Their performance is constrained by the finite number of orders and parameters of the model [22], which results in the unaligned regions that are distantly located from aligned regions in a long read losing opportunities for correction. In addition, the training phase of HMMs is quite time-consuming.

Fortunately, RNN [23] algorithms have been found to be able to effectively capture and process long-term dependencies between sequences for sequence labeling, which creates the internal hidden state of the network that allows it to exhibit dynamic temporal or spatial behavior and shines in biological sequence analysis [24]. DeepVariant [25] and Deepnano [26] both convert the problem of variant calling and base calling into classification tasks in deep learning. However, the features and labels used for training to classify are manually specified, and the one-to-one correspondence between them is also specified based on subjective experience. Meanwhile, classification models often encounter the problem of imbalance classes, and the training data needs to be adjusted based on prior knowledge. The generative sequence-to-sequence(seq2seq) [27] model can automatically solve the above limitations, enabling NMT to effectively translate text from one language to another. NMT model based on the seq2seq framework replaces the statistical machine translation (SMT) model based on HMM or SVM and becomes the latest underlying framework of Google Translate [28]. There are researchers who have begun to explore the possibility of analyzing biological sequences with NLP ideas. ProLanGO [29] converts the prediction of protein functional regions into a language translation task for the first time and reaches a high accuracy.

Thus, a possible solution to the long-read correction problem has been conceived. In this work: 1) RNN is used to overcome the limitation of HMM in capturing long-term dependencies, allowing the method to ignore the different error profiles of sequencing platforms and correct errors by leveraging the contextual relationships within long reads; 2) An NMT model is employed to identify differences between the aligned regions of long reads and the corresponding short reads for error correction; 3) A seq2seq-based generative framework is used to address the bottleneck of unequal input and output lengths in long-read error correction, a problem that cannot be solved by traditional classification tasks requiring one-to-one correspondence between inputs and outputs.

Consequently: 1) This study is the first to apply a generative language model for hybrid error correction, not only learning the discrepancies between long reads and short reads to correct errors in aligned regions, but also effectively capturing bidirectional contextual relationships to correct those often-overlooked unaligned bases; 2) This approach improved sequence quality, including alignment identity and the number of aligned bases, while maintaining length and continuity; 3) Compared to non-machine learning algorithms, it can enhance sequence quality without being platform-dependent, and compared to the only existing machine learning algorithm, it breaks through the finite state space of HMMs and capture context to fix those unaligned regions. Ultimately, it demonstrates better comprehensive performance over all other non-machine learning and machine learning algorithms.

## Methods

This part is structured into three main sections: First, long-read sequences need to be understood as natural language sequences by machine, with the original long-reads serving as the sentences from the source language and the alignment information between the original long reads and short reads serving as the corpus of the target language for translation. Second, how to construct a generative translation model to facilitate translation from the source language to the target language is discussed. Third, several key implementation details crucial for successful model fitting, including hyperparameter settings, data generator, zero-padding masking, and source sentence reversal are outlined. As a result, the well-trained NMT model with alignment information is used for error correction.

### Understanding a long read as a sentence by machine

Intuitively, there are many similarities between long-read sequences and natural language sequences, both are time-series composed of characters. To accomplish translation tasks, long-read sequences need to be transformed into a form that can be understood and processed by machines. “Understanding the alignment information” section describes how to understand the alignment information. “Generation of corpus” section explains how to generate corpus of the source and target languages from the align information. “Encode tokens to one-hot arrays” section discusses how to convert the corpus into a vector stream format that can be used for model computation.

#### Understanding the alignment information

Imagine a scenario where a model is tasked with translating the source language sentence “Je veux manger une pomme.” into the target language sentence “Ich will Äpfel essen.”. To do this, the model must grasp two key pieces of information: First, the two sentences must be semantically aligned, meaning that they both represent the concept of “I’d like to eat an apple.” Second, the elements in a sentence must be semantically correlated. Inspired by this translation mechanism, translating long reads into corrected long reads involves understanding the correspondence between the original long-read and the corrected long-read sequences, as well as the forward and backward correlations within each long-read sequence to generate a new target sequence. The former is provided by the alignment information between the long reads and homologous high-precision short reads, while the latter is derived from the long read itself.

**Fig. 1:**
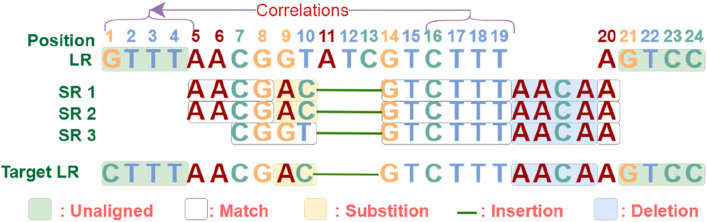
Relationship between the original long read, the aligned short reads and the target long read

When aligning short reads to a long read, it does not necessarily mean that every base in the short read perfectly corresponds to each base in the aligned region of the long read. There could be various insertions, deletions, and mismatches at any position within this region. The specific alignment pattern is represented by a CIGAR string. By parsing CIGAR strings from many aligned short reads, the coverage status of a long-read region can be determined. It is generally accepted that there is a specific correspondence between this region of the long read and these aligned short reads. Ultimately, the hybrid error correction process involves leveraging the alignment information to correct the corresponding regions on the long read, as illustrated in Fig. 1.

For example, the CIGAR string for the aligned SR1 to the long read is “5:4M2S3D6M4I1M”, indicating that starting from position 5 of the long read, there are 4 matches, 2 substitutions, 3 deletions, 6 matches, 4 insertions, and 1 match. Figure 1 is used to illustrate such correspondence between the bases of these two sequences. Similar correspondences for SR2 and SR3 are also illustrated in the figure. From the visualized alignment of the three short reads, the target long read corresponding to the original long read is generated, labeled as Target LR. When the short reads (SR1, SR2, SR3) are aligned to the long read (LR), two substitutions occur at positions 9 and 10 twice for SR1 and SR2, resulting in the bases GT in the long read to be replaced with the target bases AC. Three deletions occur at positions 11, 12 and 13 for all short reads, indicating that these positions should not contain any base, thus, the original long-read bases ATC should be removed. Four insertions occur between positions 19 and 20 for all short reads, suggesting that the bases AACA are missing in this region, and AACA should be added here to the target sequence. These changes are identified by analyzing the discrepancies within the alignment information of long-read and short-read sequences. Whereas the modification at position 1 is influenced by the forward and backward relationships among bases at positions 16, 17, 18 and 19. Because a well-trained bidirectional RNN suggests that the probability of observing CTTT in the context of AAC is higher than that of observing GTTT. The arrow in Fig. 1 indicates how the forward and reverse relationship inherent in the long-read sequence impact the correction of unaligned regions.

**Fig. 2:**
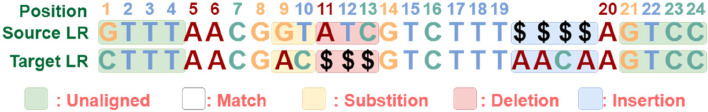
The placeholder filling process for the source and target sequences

Since the locations and the numbers of indels are irregular, the insertion or deletion of bases disrupts the alignment of subsequent k-mers, making it difficult to fit a machine translation model. Thus, it is necessary to apply simple filling of placeholders to both the source long-read sequences and the target long-read sequences. When several insertions occur in a short read aligned long-read fragment, the same number of “$” placeholders are used to fill the corresponding positions in the target read, the filled target read is referred to as the target sequence. When some deletions occur in a long-read fragment, the same number of “$” placeholders are used to fill the corresponding positions in the long read, the filled long read is referred to as the long sequence. The filling process is illustrated in Fig. 2.

#### Generation of corpus

**Fig. 3:**
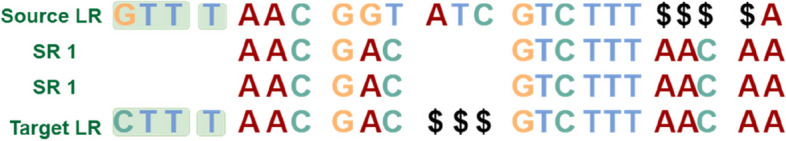
The tokenization of the long sequence and the target sequence

To capture the differences and relationships required for the afore-mentioned machine translation, the model must be trained carefully with a corpus. The training process involves using the model to capture the maximum conditional probability of a given sequence of tokens occurring. In seq2seq models, conditional probability is typically expressed as the probability of predicting the target sequence from a given source sequence. This probability is used to predict the tokens in the target sequence, one by one, based on the individual tokens in the source sequence [30]. Therefore, the source and target sequences should be tokenized first. In the current task, tokenization refers to the segmentation of source and target sequences generated in the previous subsection. First, each sequence is segmented into adjacent but non-overlapping k-mers according to a fixed length k. All these k-mers with the specific order form the corpus. Although overlapping k-mers are often used in DBG-based error correction algorithms to preserve the contextual correlation between sequences, overlapping k-mers will increase the size of the corpus, which will consume more computing resource. Meanwhile, when the model achieves high enough accuracy by training, the model will be able to automatically capture the contextual correlation between k-mers, thus there is no need to use overlapping k-mers. Figure 3 is a simple segmentation process of a long read with 3-mers. Since the sequence contains 5 kinds of characters {ATGC$}, the maximum size of the vocabulary is 5^k, and the target sequences are processed in the same way to get the target vocabulary.

In Fig. 3, the green unaligned region and the transparent aligned region are segmented into tokens of length 3 respectively. The objective of model training is that: given the known token AAC, the model assigns the highest probability to the token GAC as the next token. Then, given both tokens AAC and GAC, the model estimates that the most likely token to occur next is $$$, and this process continues until the entire target sequence is generated.

**Fig. 4:**
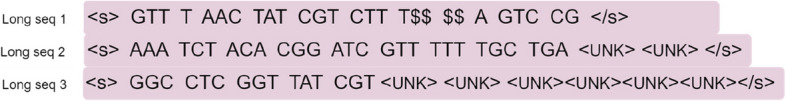
Padding process. There are 13 tokens in the padded sequences

It is worth noting that the length of each long sequence is unequal, the number of tokens of each sequence obtained after tokenization is also unequal. However, during seq2seq training, it is required that each batch has the same data shape, meaning that the number of tokens in each sentence must be consistent. Otherwise, the generation for the current batch will stop upon encountering the earliest ‘end-of-sequence’ character ‘ < /s > ’ in that batch. The specific operation is as follows, 1) all sequences are traversed to get the maximum number of tokens, and then all sequences that do not reach the maximum length are padded with a token ‘ < UNK > ’. 2) The start token ‘ < s > ’ is added at the beginning of each sequence, 3) and the end token ‘ < /s > ’ are added at the end of each sequence. The start token ‘ < s > ’ is used to inform the model to start prediction, and the end token ‘ < /s > ’ is used to inform the model that the prediction should be terminated. It is worth noting that, during the model training process, the calculation of the loss of the padded tokens needs to go through a special zero-value mask to remove irrelevant predictions. The specific calculation method will be discussed in detail in the next section. Assuming that the maximum length of long-read sentences is 13, the padding process is shown in Fig. 4.

#### Encode tokens to one-hot arrays

**Fig. 5:**
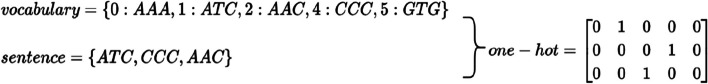
The generation of one-hot matrix. In the matrix, each row of the matrix corresponds to an array of a “word”

To enable machines to learn from the generated corpus, the input of the neural network must be a vector or matrix of numerical type, a common operation is one-hot encoding. For a given vocabulary, each token is represented as a unique vector whose dimension is equal to the vocabulary size. The component in the vector is 1 at the index position of the corresponding token and 0 at other positions. For example, assuming a vocabulary size of 10,000, the word “apple” is located in the 1000th position in the vocabulary, it can be represented as a 10,000-dimensional vector with only the 1,000th position being 1 and the other positions being 0. This kind of vector is called a one-hot vector. In neural machine translation, the source sequence and target sequence are usually composed of multiple tokens. Therefore, each token in the sequence needs to be represented as a unique one-hot vector, and the entire sequence is represented as a matrix where each row contains a one-hot vector of a token. The advantage of using a one-hot matrix is that it can completely represent the discrete relationship between tokens and can be directly used for calculations in neural networks. A simple one-hot matrix generation is shown in Fig. 5. In the same way, the target vocabulary belonging to the target “sequences” is generated, and the one-hot matrix of each “target sentence” is obtained in the same way.

Although the one-hot matrix is sparse, it can retain all important information in the sequence. The entire sequence can be restored by searching the one-hot index in the vocabulary. The dimensionality reduction techniques used in natural language to save space are not applicable in our scenario, such as word2vec and embedding [31]. The reason is that dimensionality reduction based on the attention of semantics and grammars. In the corpus of long “sentences”, there is no obvious semantic or grammar founded so that the vectors obtained after embedding tend to be uniformly distributed. This kind of vectors will increase the number of iterations during training, and the complex embedding process also consumes the running time and computing resource without any performance improvement. In addition, any dimensionality reduction technique would discard some information of original data. Therefore, no embedding is needed here. So far, the long reads are converted into numerical values that can be understood by the machine.

### The construction of neural machine translation model

Once the long reads are converted into vectors understandable by machines, the next task is to build a model that can learn the required information. To overcome the inconsistency in length between before- and post-corrected long reads, an end-to-end translation model that can ignore the length disparities in input and output is constructed, namely, seq2seq model. Seq2seq models allow the model to map between source and target sequences without requiring handcrafted rules. They can also handle variable-length sequences, both on the input and output sides. This flexibility is crucial for the current error correction task, where the input long reads have varying lengths with their corresponding corrected reads.

Furthermore, RNN is considered as a general technique that can effectively capture long-term dependencies within long sequences. As an efficient variant of RNN, Bi-LSTM is used here as the core layer of the model. Finally, to ensure that the model does not overfit during the training process, reasonable data segmentation for the input datasets is implemented here.

#### The architecture of seq2seq

In the seq2seq framework, the encoder can transform the input long sequence of variable length to a fixed-length context vector *C*. RNN layers, such as Gated Recurrent Unit (GRU) [32], LSTM, are usually used within the encoder. To continuously generate tokens of the output sequence, another recurrent neural network predicts the next token according to both the encoded information of the input sequence and the tokens and states generated from the output sequence previously.

**Fig. 6:**
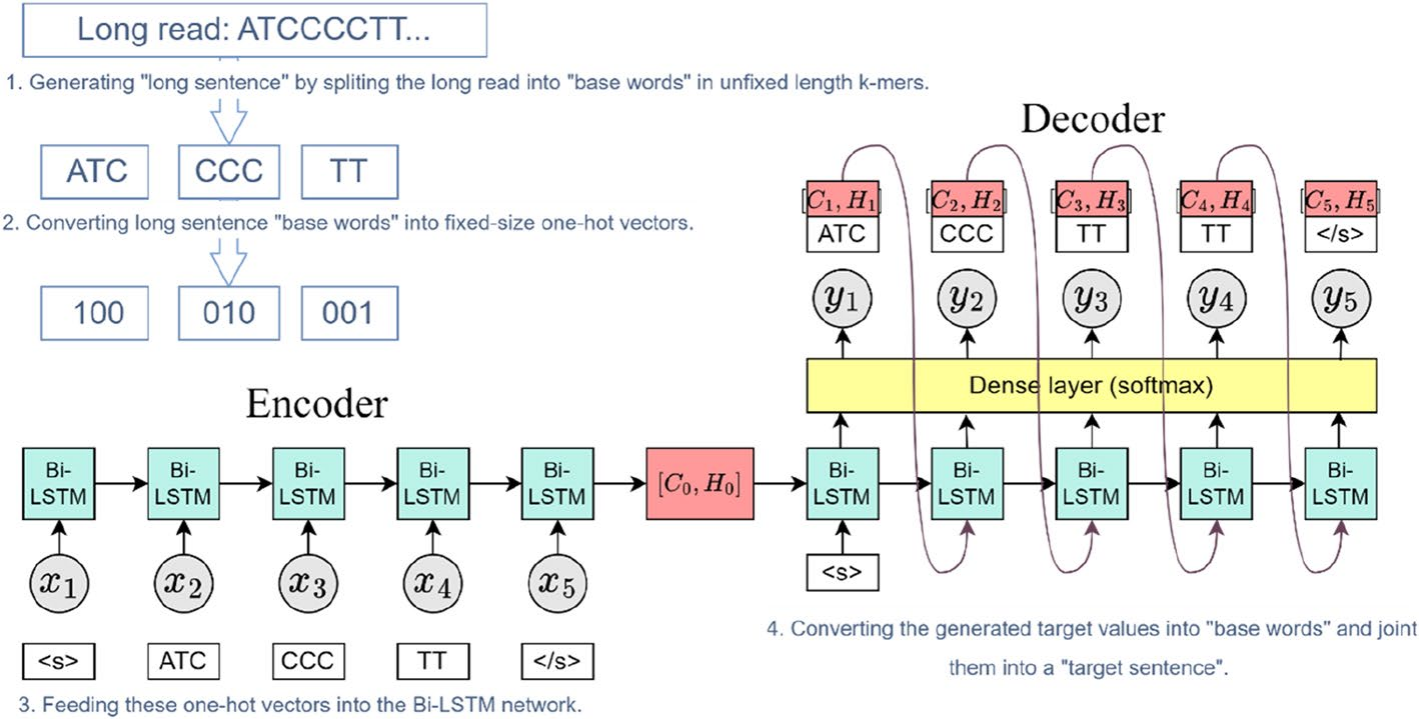
Flow chart of sequence prediction by the proposed framework

Suppose that there is an input sequence ${x}_{1},\dots ,{x}_{T}$, where ${x}_{t}$ is the ${t}^{th}$ word. At time step *t*, the Bi-LSTM will save the token ${x}_{t}$ and the hidden state of the last time step *h*_*t*-1_. Next, the encoder captures information of hidden states and tokens from all of the time steps and encodes them into the context vector *C*. Suppose that the given outputs in the training set are ${Y}_{{1}^{\prime}},\dots ,{Y}_{{T}{\prime}}$. At each time step $t{\prime}$, the conditional probability of output ${Y}_{{t}^{\prime}}$, $P\left({Y}_{{t}{\prime}}\left|{Y}_{{1}^{\prime}},\dots ,{Y}_{{t-1}^{\prime}},C\right.\right)$, will depend on the previous output sequence ${Y}_{{1}^{\prime}},\dots ,{Y}_{{t-1}^{\prime}}$ and the context vector $C$. To model this conditional probability, another Bi-LSTM network is used as the decoder. At time step $t^{\prime}$, the decoder will update its hidden state according three inputs: the feature vector from last time step ${Y}_{{t-1}^{\prime}}$, the context vector C, and the hidden state of last time step ${h}_{{t-1}^{\prime}}$ After obtaining the hidden state ${h}_{{t}^{\prime}}$ by the decoder, the *softmax* function of the output layer is used to calculate the conditional probability distribution of the output at time step $t^{\prime}$, and then solve the output token of time step $t^{\prime}$. It is worth noting that when implementing the decoder, the hidden state of the encoder in the final time step is used directly as the initial hidden state of the decoder. This requires that the encoder and decoder Bi-LSTM layer have the same number of hidden units. The only difference between encoder and decoder is that a dense layer after the Bi-LSTM layer is needed in the decoder to predict the maximum probability for each token, and the number of units in the dense layer is the same as the target vocabulary size. The flow chart for sequence prediction of the proposed framework is shown in Fig. 6.

As described in the figure, the encoder and decoder are built with a Bi-LSTM layer. The input long sequence is decomposed into tokens ${x}_{1}, {x}_{2}, {x}_{3}, {x}_{4}, {x}_{5}$ and fed to the encoder, whose sole purpose is to create the context vector ${C}_{0}$ and return the hidden state vector ${H}_{0}$. The output of the encoder $\left[{C}_{0}, {H}_{0}\right]$ is regarded as the initial state of the decoder and the specific start token ‘ < s > ’ is sent to the decoder to start predicting the output token. Thereafter, the token generated at the current time step and the updated hidden state vector become the input of the next time step, prompting the decoder to predict the output token of the next time step. Once the model generates end token ‘ < /s > ’, the model will stop prediction.

#### The selection of encoding and decoding layer

**Fig. 7:**
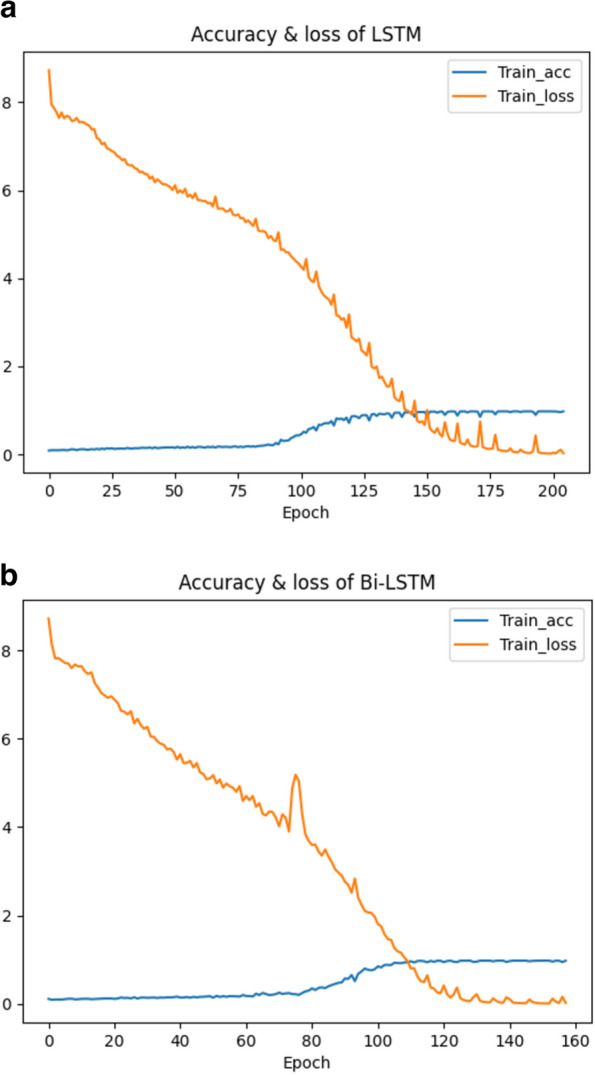
The change of loss and accuracy with epoch for LSTM and Bi-LSTM. **a** The change of loss of LSTM with epoch. **b** The change of loss of Bi-LSTM with epoch

The following section will further explain why the afore-mentioned seq2seq model chooses Bi-LSTM as its core layer and how it works. LSTMs are designed to handle long-range dependencies and remember information over time due to their unique cell structure with memory cells and gating mechanisms. Bi-LSTMs process input sequences from both forward and backward directions. This is valuable in seq2seq models because the context in a sequence does not always flow in one direction. By processing in both directions, Bi-LSTMs can capture context and dependencies more comprehensively. As a result, Bi-LSTMs are used in the encoding phase to ensure that the encoder captures the full context of the input sequence. This context is then passed to the decoder, which generates the output sequence. The accuracy of LSTM and bi-LSTM changes with the number of epochs as shown in Fig. 7.

When LSTM is used as the encoding layer, the loss starts to converge to 0 when the training iterates about 200 times, and the accuracy starts to converge to 1 when the iterates about 125 times. While, when Bi-LSTM is used as the encoding layer, the loss starts to converge to 0 when the training iterates 120 times, and the accuracy starts to converge to 1 when the training iterates about 100 times. The model with Bi-LSTM fits more quickly during training.

#### Dataset segmentation

Data segmentation involves splitting a dataset into training, validation, and testing sets distinctively. The training set is used to train the model, the validation set helps tune hyperparameters and prevent overfitting, and the test set is used to evaluate the final performance of the model. Suitable segmentation can improve training efficiency and generalization. In the current application scenario, the hash algorithm that hashes sequence names is employed to randomly shuffle the long-read sequences when creating the corpus, ensuring that each dataset has randomness and representativeness. Given that the long-read datasets are relatively large, the proportion of the training set is increased, Hence, the 80% is the training set, 10% is the validation set, and 10% is the test set. Through observation, the model converged effectively on the training set without overfitting, and it performed well on the test set, indicating that this data segmentation is reasonable and effective.

### Implementation details

So far, the network has been built and the long-read sequences are transformed into a format that can be fed into the network. However, several key details are crucial for rapid and effective model convergence, including hyperparameter settings, data generators, zero-padding masking, and source sentence reversal.

#### Hyperparameter settings

The choice of hyperparameters directly affects the performance and generalization ability of the model. An overly complex model may lead to overfitting, while an overly simple model might result in underfitting. Different hyperparameter combinations are tested and set manually as follows:

1. Neurons in Bi-LSTM: Too few neurons may not be able to extract sufficient information, while more neurons can better capture complex correlations. However, too many neurons increase training time and risk overfitting. The model with Bi-LSTM for both the encoding and decoding layers with 256*2 neurons converges the fastest without causing GPU overflow. Additionally, the dense layer in the decoder is used for classification, with the number of categories corresponding to the size of the target vocabulary. Thus, the number of neurons in the dense layer is set to the size of the target vocabulary.
2. Learning Rate and Optimizer: The initial learning rate is set to 0.1, combined with the Adam optimizer, which can adjust the learning rate according to the gradient descent.
3. Batch Size: Given the model’s fitting condition and hardware constraints, the batch size is set to 64.
4. Early Stopping: Instead of a fixed number of training epochs, we use early stopping to prevent overfitting. The training will stop early if the model does not improve within 5 iterations (patience = 5).

#### Data generator

Since there are too many “long sentences” to be processed by the model at one time, the datasets should be split to facilitate the processing of the model, thus, a data generator is used to split the dataset into small batches to feed into the model, reducing the memory usage and preventing GPU overflow. For the proposed scheme, the goal is to predict the next token in the target sequence based on the tokens observed so far in the source sequence, the label is the next token in the target sequence. Therefore, using the method of sequential partitioning to load data in small batches can preserve the correlation between sequences as much as possible and improve the performance of the model. The order of split subsequences should be preserved during iteration, ensuring that the subsequences from two adjacent small batches are also adjacent in the original sequence.

Actually, the weights of the model will be updated for each batch of data during the training. It is also necessary to predict the output values for a batch of data. Since, if the model trained in batches is used for the prediction of an entire sequence, there will be prediction bias. It should be noted that the hidden states of each time step should be updated and fed into the next time step for prediction. For example, if the batch size of the data is 64, the model will predict one token for each of the 64 sequences at the same time and update 64 hidden states accordingly. These 64 hidden states should be used during the prediction of the next time step.

#### Zero-padding masking

Since the Keras library requires the tokenized source sequences in a batch should be the same length, the token ‘ < UNK > ’ is used to pad the shorter sequences, ensuring that all sequences in a batch have the same length. Note that the tokenized target sentences are also padded to make them have the same length, but there is no need to compute the loss on the padded symbols. An operation referred to as “Sequence Mask” is adopted to remove the token ‘ < UNK > ’ in the calculation of the loss. Specifically, the masks of all the actual tokens are set to 1 and the masks of the token ‘ < UNK > ’ are set to 0, and the loss matrix is multiplied by this mask matrix to get the actual loss. In this way, the model has filtered out irrelevant predictions produced by the padding tokens. The padded token including ‘ < s > ’ and ‘ < UNK > ’ should be removed after prediction.

#### Source sentence reversal

**Fig. 8:**
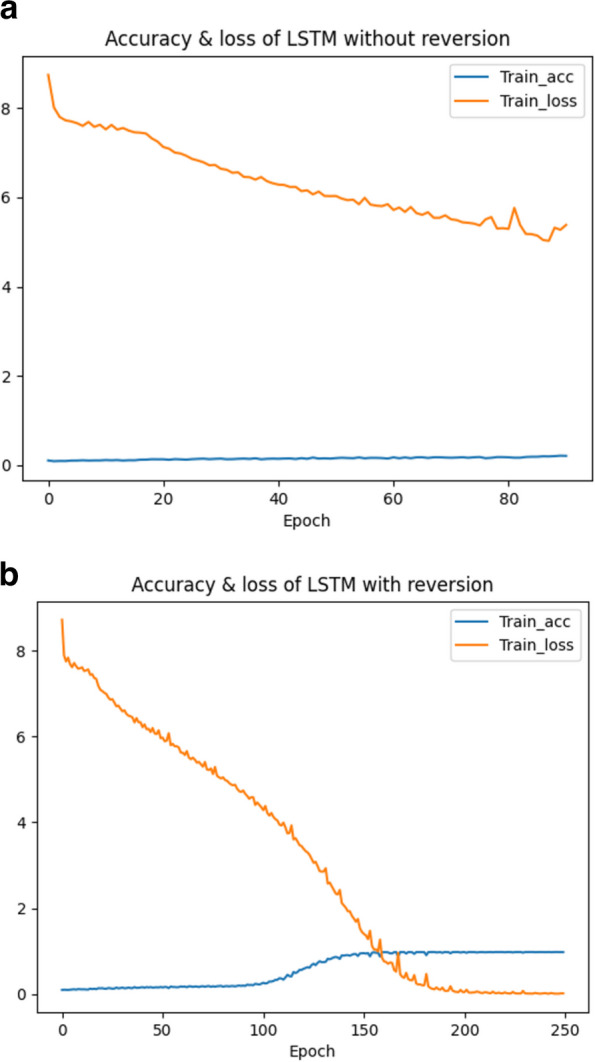
The convergence of loss with or without source sentence reversal. **a** The convergence of loss without reversal. **b** The convergence of loss with reversal

To establish better communication between the “source sentences” and the “target sentences”, the order of the tokens in the input sequence is reversed, which is referred to as source sentence reversal. The main intuition behind reversion is that by reversing the order of the input sequence, the model receives the final tokens first. The hidden state ${H}_{t}$ of the last time step output by the encoder is sent to the decoder to become the initial hidden state ${h}_{0}^{\prime}$, which is used, together with the context vector c from the encoder, to predict the output of the first token. At this point, the source sentence reversal technique can fully correlate the last token of the encoder with the first token of the decoder. Specifically, instead of using the sequence $a,b,c$ to predict $\alpha ,\beta ,x$, where $\alpha ,\beta ,x$ is the translation of $a,b,c$, we use $c,b,a$ to predict $\alpha ,\beta ,x$. In this way, the last hidden state from the encoder (for a) is sent to the decoder and used as the initial hidden state for predicting $\alpha$. After that, the previous hidden state (for b) is received by the decoder to predict $\beta$. Such a simple trick of reversing the order of the input tokens in the “long sentences” can effectively make the model converge. Another benefit of source sequence reversal is that the “source sentence” ends with the token ‘ < s > ’ which is the same as the token that the “target sentence” starts with, thus, there is no need to perform another processing to build the connection between the end of the “source sentence” and the start of “target sentence”. After generating “long sentences”, the order of each token in a “long sentence” is reversed before this sequence is fed into the network. Figure 8 shows the convergence of the loss with or without source sentence reversal.

As shown in Fig. 8a, the model stops training when the loss reaches around 5.5 and not decreases anymore. The excessive loss means that the model training has failed. As shown in Fig. 8b, the loss converges to 0 after training, indicating that source sentence reversal can greatly boost the performance of the proposed scheme.

## Transfer learning

As of now, the model described previously can correct a small long-read dataset. 17 sequences are sampled from the *E.coli* data set randomly and used the above NMT model to conduct correction. The result shows that the number of bases and alignment identity corrected by NmTHC are prominent to other mainstream methods. However, the generative sequence model is quite time-consuming. It takes more than 20 min to correct a small dataset of only 150KiB. Extrapolating this, it would take 70 days to correct a complete E. coli long-read dataset using a single GPU, which is intolerable. Fortunately, there are many obvious similarities among long-read sequences and within an individual sequence, which is also can be seen in the high-frequency tokens. In previous training processes of generative models, the features of the entire long-read data were divided into batches of the size ‘*batch_size*’ and sequentially fed into the network for training, with each batch serving as the smallest unit for parameter updates. This means when a new set of data is input into the network, the network will begin updating parameters from the start. Such a training strategy neglects the similarity between long-read sequences and results in a high learning cost. At this point, transferring the learned parameters from similar structures to the entire training process of long reads would greatly reduce learning time. Thus, a model-based transfer learning strategy is employed to learn and transfer these similar regions to address the time-consuming issue of the correction model. Specifically, a pre-trained model is used to extract these similar structures from the source domain and share them through parameter transfer. Then, the obtained model is fine-tuned, such as reducing the learning rate or changing the loss function. Finally, conduct supplementary training with target domain data that is similar to the source domain, which allows a fast fitting for a higher-precision model.

The little similarity is likely to result in the negative transfer, which means the model would not fit in the target domain. On the other hand, too much similarity may lead to a low a generalization performance of the model. Therefore, how to divide the long-read dataset with reasonable similarity has become the key to the current transfer task. Fortunately, the long reads with similar name generally come from DNA fragments close to each other, and these long reads usually have high degree of similarity. Therefore, each sequence name is hashed first, and sequences with the same hash value are placed in the same chunk. The entire dataset is randomly divided into N chunks. N-1 is the number of times the model needs to be transferred in the future. N can be adjusted according to the size of the dataset and the change of the loss after transfer. *N* = 500 or 1000 is recommended here. There should be noted that if the chunks are too small, it is not possible to capture the global structural similarities to get a satisfied loss and accuracy in the fine-tuning stage. On the other hand, if the chunks are too large, it would result in an over-sized vocabulary for the dense layers to predict, the efficiency of transfer learning would be affected.

**Fig. 9:**
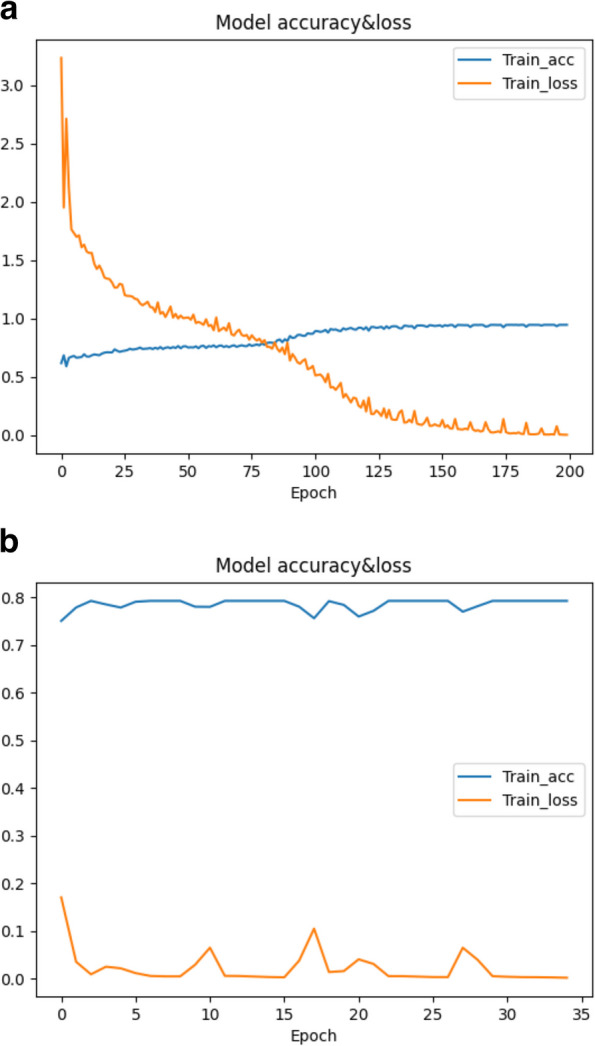
The change of loss and accuracy during the transfer learning process. **a** Model convergence in the pre-training stage. **b** Convergence of the model in the fine-tuning stage

After dividing step, there are two steps for transfer: 1) Pre-training and saving the parameters: choose any chunk for pre-training and save the weights of each layer when loss and accuracy are high enough; 2) Unfreezing and fine-tuning: Unfreezing is making the previously frozen neural network layers trainable so that the model can update the parameters of these layers on a new task to adapt to the target data. In the current work, a lower learning rate is applied for the fine-tuning of the model. We divide the fruit fly long-read dataset according to *N* = 10,000. There are 48 sequences in the first chunk, and the number of bases is 540955 bp. The loss and accuracy curves of pre-training and fine-tuning after successful transfer are shown in Fig. 9a and b. The results show that the model requires 175 epochs to obtain satisfactory loss in the pre-training stage, but when the pre-trained model is used as the initial weights after transfer, it can be fitted in only 35 epochs to get the low enough loss for error correction.

## Results

### Performance of model training

**Fig. 10:**
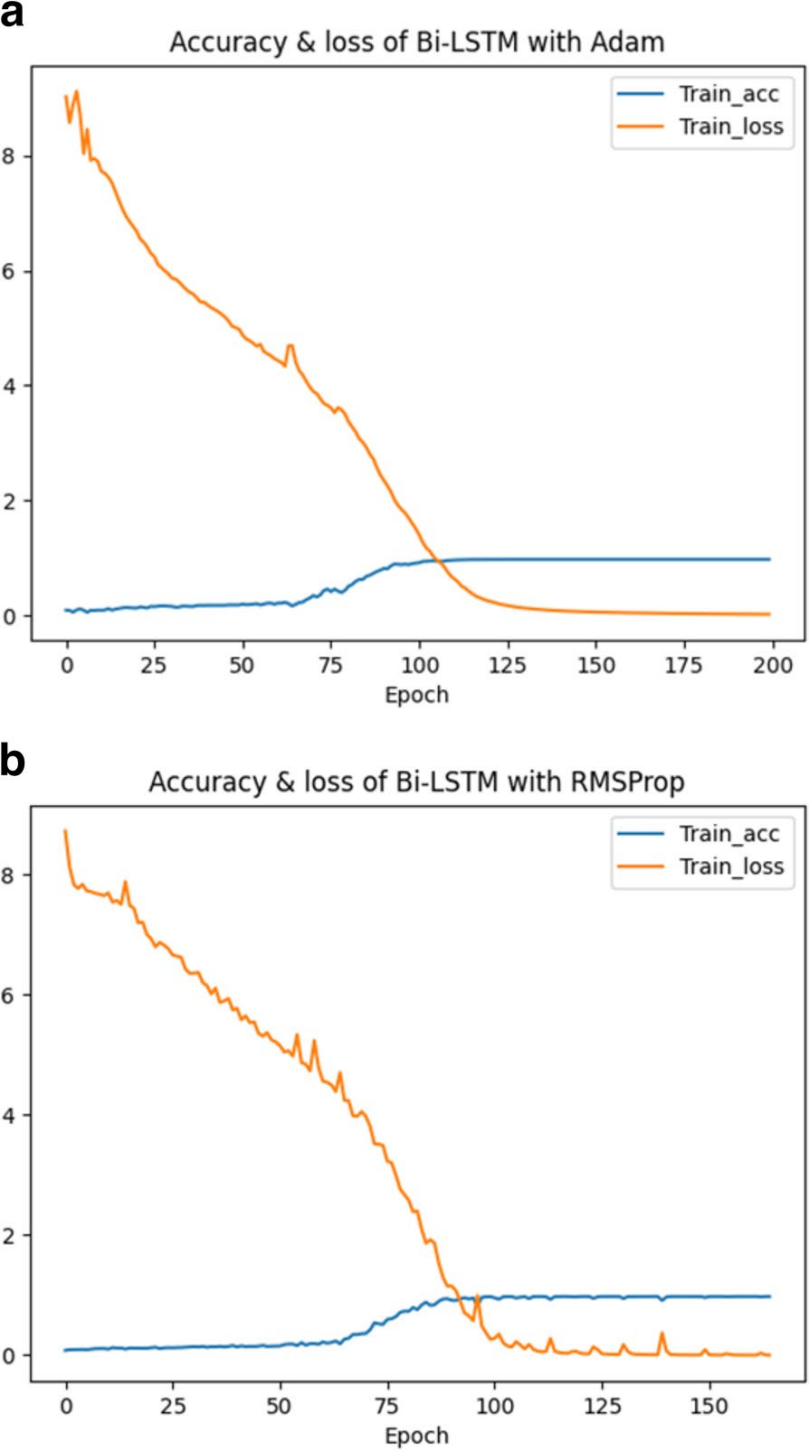
The changes of loss with the number of epochs when different optimizers are used. **a** Loss gradient when Adam is used as the optimizer. **b** Loss gradient when RMSProp is used as the optimizer

During the training process, the cross-entropy classification function is used to calculate the loss of the model, and accuracy is used to evaluate the model. Increasing the batch size can accelerate training as it reduces the number of time-consuming computational backpropagations [33]. However, the excessively large batch size would not only cause GPU overflow but also increase the possibility of the model stopping at a local minimum. Meanwhile, too many neurons in the Bi-LSTM network also makes GPU overflow. Therefore, the batch size is set to 64, and the number of Bi-LSTM neurons is set to 512. Adaptive moment estimation optimizer (Adam) [34] and root mean square prop optimizer (RMSProp) [35] are tested under the same conditions. The results from Fig. 10 show that Adam can make the loss converge more smoothly. Thus, the Adam algorithm is chosen as the optimizer. The maximum number of epochs for model training is set to 200, and the early stop mechanism with patience 5 is set to cut off the training when the loss does not decrease within 5 iterations. In this way, the model can be effectively prevented from overfitting. The results of the model training show that the loss of the model converges to 0 and the accuracy converges to 1 after about 130 iterations, indicating that the training of the model is successful.

### Datasets and experiment setup

The six datasets used in our experiments are from three species, Escherichia coli K-12 MG1655 (E. coli), Saccharomyces cerevisiae S288C (yeast), and Drosophila melanogaster ISO1 (fruit fly), from three platforms, Pacific bioscience (PacBio), Oxford Nanopore (ONT) technologies, and Illumina. For each dataset, the long reads to be corrected are from the PacBio or Nanopore platforms, and the high-quality short reads that are used to correct long reads are from the Illumina platform. However, the availability of reference genomes of these strains enables us to evaluate the correction quality in a reliable manner, and they are sequenced and assembled carefully by Sanger and other institutions. The details of the datasets are listed in Table 1.

**Table 1:** Information on the selected datasets

Sequencing specification	Sequencing NCBI accession	Number of reads	Reference genome	Genome length(Mbp)	Reference NCBI accession
Illumina Miseq	_a	2 × 5729470	E. coli K-12 MG1655	4.6	NC_000913.3
PacBio P6C4	_b	87217			
MinION R9 1D	_c	164472			
Illumina Miseq	ERR1938683	2 × 3318467	S. cerevisiae S288c	12.2	GCF_000146045.2
PacBio P6C4	PRJEB7245	239408			
MinION R9 2D	ERP016443	119955			
Illumina Nextseq	SRX3676782	2 × 20 619 401	Drosophila melanogaster ISO1	143.7	GCF_000001215.4
Pacbio P5C3	SRX499318	6864972			
MinION R9.5 1D	SRX3676783	663784			

^a^Downloaded from Illumina at ftp://webdata:webdata@ussd-ftp.illumina.com/Data/SequencingRuns/MG1655/MiSeq_Ecoli_MG1655_110721_PF_R1.fastq.gz and ftp://webdata:webdata@ussd-ftp.illumina.com/Data/SequencingRuns/MG1655/MiSeq_Ecoli_MG1655_110721_PF_R2.fastq.gz with browser or wget command in a Linux environment

^b^Downloaded from PacBio at https://github.com/PacificBiosciences/DevNet/wiki/E.-coli-Bacterial-Assembly

^c^Downloaded from Loman Labs at https://s3.climb.ac.uk/nanopore/E_coli_K12_1D_R9.2_SpotON_2.pass.fasta

NmTHC and 7 other typical hybrid error correction algorithms, including LoRDEC, Jabba, FMLRC, ColorMap, HALC, Proovread and Hercules, are used to correct the obtained datasets, and their results are compared. The command line parameters of each algorithm are based on the manual provided by respective author, and the details are recorded in Table 2. All experiments in this work are run on a server with 2 CPU (Intel Xeon Gold 6240 @ 2.60 GHz 72 cores), 256 GB memory, and 2 GPUs (Quadro RTX 6000, Compute Capability 7.5). For NmTHC, the process of alignment and tokenization is implemented on the CPU, the training and prediction of the model are implemented on the GPU. All source codes are based on Python 3.6 and TensorFlow-gpu 2.3.

**Table 2:** The command line parameters of each algorithm

Method	Command line parameters
LoRDEC	LoRDEC-correct -2 short_reads.fasta -k 29 -s 3 -i long_reads.fasta -o long_reads_LoRDEC.fasta
Jabba	karect -correct -matchtype = hamming -celltype = haploid -inputfile = short_reads.fasta Jabba -o Jabba_output -k 75 -t 64 -g brownie_output/DBGraph.fasta -fasta long_reads.fasta
FMLRC	time gunzip -c short_reads.fq.gz | awk 'NR % 4 = = 2' | sort | tr NT TN | ropebwt2 -LR | tr NT TN | fmlrc-convert -f./output/comp_msbwt.npy fmlrc -k 21 -K 59 -p 1./output/comp_msbwt.npy long_reads.fasta fmlrc_long_reads.fasta
ColorMap	runCorr.sh long_reads.fasta short_reads.fasta ont pre 16
HALC	ABYSS short_reads.fasta -k 21 -o output python runHALC.py long_reads.fasta contigs.fa -t 64 -o short_reads.fasta
Proovread	Proovread -l long_reads.fasta -s short_reads.fasta --pre Proovread
Hercules	Hercules -2 -li long_reads.fasta -ai sorted.bam -si short_reads.fasta -t 30 -o hercuels_corrected.fa

### Performance evaluation indicators and results

As described by LRECE [36], the biggest of the error correction algorithm is absence of ground truth (i.e., perfectly corrected reads). Fortunately, the reference genomes can evaluate these algorithms reliably. Essentially, the differences between the corrected long reads and the reference genome mean uncorrected errors. In this way, the quality of error correction can be obtained by evaluating the quality of the alignment of the corrected sequence to the reference genome. In practice, Minimap2 [37] is used to align both the original and the corrected long reads to their reference genome with the command line “*minimap2 -x map-pb/ont -t 30*”. Finally, various performance indicators of these alignments are calculated to evaluate the error correction performance of the algorithms. The experimental results are calculated by LRECE. The results on sampled E. coli datasets from two platforms are shown in Tables 3 and 4, the results on sampled yeast from two platforms are shown in Tables 5 and 6, and the results on sampled fruit fly from two platforms are shown in Tables 7 and 8.

**Table 3:** Experimental results for the E.coli PacBio dataset

Method	Total bases	Aligned bases	Alignment identity	Average length (bp)	Maximum length (bp)	N50 (bp)	Usr time (m:s)	Memory usage (GiB)
Original	748009625	729784022	0.9756	8752	44113	13990	--	--
Short-read-DBG-based methods								
LoRDEC	716893126	702098565	0.9793	8402	44133	13491	1332:56	4.8
Jabba	611947598	611947598	1	7880	41342	12352	161:55	2.1
FMLRC	748004466	719532482	0.9619	8752	44117	13400	225:45	19.5
Short-read-alignment-based methods								
ColorMap	730726602	715441895	0.9790	8529	44113	13641	1728:22	24.5
PR-trim	537183316	537132179	0.9990	4971	39836	9435	2804:4	7.5
PR-untrim	607114493	593125,625	0.9769	9786	44113	14559	2804:4	7.5
Short-read-assembly-based methods								
HALC	711074601	698299377	0.9820	8340	44136	13400	27357:4	4.0
HALC-trim	689081519	683004708	0.9911	8250	44066	13222	27357:4	4.0
HMM-based method								
Hercules	742217998	723994674	0.9754	8691	44113	13887	95964:55	4.8
Deep learning-based method								
NmTHC	743904487	743534260	0.9995	8723	44113	13941	2656:5	3.0

“PR-trim” represents the results provided by Proovread default, the long read after correction is in fact high-precision long-read fragments when the low-quality regions are trimmed. “PR-untrim” stands for the results recovered by the third-party tool “*seq*tk”. “HALC” and “HALC-trim” are both results provided by HALC default

**Table 4:** Experimental results for the E.coli ONT dataset

Method	Total bases	Aligned bases	Alignment identity	Average length (bp)	Maximum length (bp)	N50 (bp)	Usr time (m:s)	Memory usage (GiB)
Original	1481511788	1479176967	0.9984	9047	131969	14895	--	--
Short-read-DBG-based methods								
LoRDEC	1555452836	1555128350	0.9997	9493	137887	15664	3044:28	4.8
Jabba	1258239439	1258239439	1	7709	93396	12436	137:49	2.1
FMLRC	1481511784	1480251346	0.9991	9047	131969	14895	363:48	19.5
Short-read-alignment-based methods								
ColorMap	1518333301	1516962292	0.9990	9253	134311	15180	1811:59	22.7
PR-trim	979107621	979107621	1	1378	28387	1662	9765:59	7.0
PR-untrim	1533953584	1532903029	0.9993	9361	137377	15419	9765:59	7.0
HMM-based method								
Hercules	1488092513	1485766466	0.9984	9087	132948	14974	136645:53	4.8
Deep learning-based method								
NmTHC	1483084718	1482913949	0.9998	9057	132122	14913	2423:46	3.0

There is no HALC correction result for the ONT dataset in Table 4, 6 and 8 because HALC is designed for PacBio SMRT long reads

**Table 5:** Experimental results for the yeast PacBio dataset

Method	Total bases	Aligned bases	Alignment identity	Average length (bp)	Maximum length (bp)	N50 (bp)	Usr time (m:s)	Memory usage (GiB)
Original	5499119594	4853379662	0.8825	9108	94868	18406	--	--
Short-read-DBG-based methods								
LoRDEC	5350446756	4885916448	0.9131	8867	94872	17925	7783:21	4.8
Jabba	2192986588	2183714060	0.9957	8501	46975	12780	990:47	2.1
FMLRC	5499317944	4834874374	0.8791	9107	94868	18406	665:48	19.5
Short-read-alignment-based methods								
ColorMap	5506697225	4860976804	0.8837	9120	94868	18434	3450:20	6.5
Proovread	--	--	--	--	--	--	--	--
Short-read-assembly-based methods								
HALC	5328432720	5027096412	0.9434	8759	94877	17840	72872:35	4.0
HALC-trim	4443811376	4300960212	0.9678	8460	57580	15662	72872:35	4.0
HMM-based method								
Hercules	5494486747	4848765700	0.8824	9102	94868	18392	202667:27	5.0
Deep learning-based method								
NmTHC	5466924180	5286441779	0.9669	9025	94868	18355	5501:5	3.0

There is no Proovread results in Tables 5 since the dataset of yeast PacBio is pair-ended sequencing data, and the long-read names are repeated, which is not allowed during the processing of Proovread

**Table 6:** Experimental results for the yeast ONT dataset

Method	Total bases	Aligned bases	Alignment identity	Average length (bp)	Maximum length (bp)	N50 (bp)	Usr time (m:s)	Memory usage (GiB)
Original	382389287	376989685	0.9858	9186	56477	11696	--	--
Short-read-DBG-based methods								
LoRDEC	390792227	386323262	0.9885	9387	58298	11966	374:2	4.8
Jabba	288736216	288726754	0.9996	7993	47266	10719	115:3	2.1
FMLRC	382297060	376708825	0.9853	9184	56477	11694	232:11	19.5
Short-read-alignment-based methods								
ColorMap	385129056	379728102	0.9859	9245	56785	11775	1461:6	2.4
PR-trim	100640	90149	0.8957	602	1102	599	513:27	7.5
PR-untrim	380864789	376072021	0.9874	9188	55897	11656	513:27	7.5
HMM-based method								
Hercules	383933798	378533253	0.9859	9223	57481	11748	64005:15	3.2
Deep learning-based method								
NmTHC	381328904	380822709	0.9986	9192	56783	11708	1738:14	3.0

**Table 7:** Experimental results for the fruit fly PacBio dataset

Method	Total bases	Aligned bases	Alignment identity	Average length (bp)	Maximum length (bp)	N50 (bp)	Usr time (m:s)	Memory usage (GiB)
Original	277577924	163084772	0.5875	2371	54186	12627	--	--
Short-read-DBG-based methods								
LoRDEC	274218615	190615200	0.6951	2345	54151	12355	1111:13	4.8
Jabba	68570230	68562572	0.9998	4980	31567	7700	2142:49	2.1
FMLRC	277373140	161807007	0.5833	2369	54184	12597	245:2	19.5
Short-read-alignment-based methods								
ColorMap	275086625	168238015	0.6115	2349	53985	12369	6139:10	2.4
PR-trim	132095964	132095964	1	4600	30412	8617	4102:13	7.5
PR-untrim	271685285	169539136	0.6440	2324	53681	12182	4102:13	7.5
Short-read-assembly-based methods								
HALC	272524283	230432806	0.8455	2331	54196	12204	2444:7	4.0
HALC-trim	221794779	202071143	0.9110	2817	52030	13237	2444:7	4.0
HMM-based method								
Hercules	275620916	162943476	0.5911	2354	54186	12420	21157:17	3.2
Deep learning-based method								
NmTHC	275927399	189503829	0.6867	2361	53282	12522	1800:59	3.0

**Table 8:** Experimental results for the fruit fly ONT dataset

Method	Total bases	Aligned bases	Alignment identity	Average length (bp)	Maximum length (bp)	N50 (bp)	Usr time (m:s)	Memory usage (GiB)
Original	4609479994	4193853794	0.9098	7177	446050	11956	--	--
Short-read-DBG-based methods								
LoRDEC	4656943723	4336450357	0.9311	7243	447498	12082	15516:57	4.8
Jabba	2277474552	2277352751	0.9999	3962	47190	6081	11999:4	2.1
FMLRC	4606352370	4110500003	0.8923	7172	444617	11949	3097:5	19.5
Short-read-alignment-based methods								
ColorMap	4685641775	4290298819	0.9156	7226	444791	12046	5338:54	2.4
Proovread	--	--	--	--	--	--	--	--
HMM-based method								
Hercules	4615570873	4202349986	0.9104	7185	446013	11974	198745:30	3.2
Deep learning-based method								
NmTHC	4605772604	4559772769	0.9900	7178	445920	11962	18943:10	3.0

There is no Proovread results in Tables 8. Proovread produces no result after 15 days of computation on our experiment platform with 72 cores, thus the process is terminated

In the experimental results, “Total bases” is the total number of bases of the long read after corrected. “Aligned bases” is the number of corrected bases that can be aligned to the reference genome. “Alignment identity” represents the consistency of the segments in the long reads and the corresponding aligned fragments in the reference genome, which is defined as the number of aligned bases divided by the total number of bases and usually inversely proportional to “Total bases”. In terms of DNA data processing, if the read is long enough, there is no need for polymerase chain reaction (PCR) amplification, which can avoid base bias and simplify genome assembly. Thus, we also compared the length of long reads after correction. “Average length (bp)” and “Maximum length (bp)” are the average and maximum lengths of regions where long reads can be aligned to the reference genome respectively. “N50” is used for assessing the quality and continuity of a sequence assembly, representing the length at which half of the entire assembly consists of sequences of this length or longer. “Memory usage (GiB)” is the peak CPU memory occupied by each algorithm during the correction.

The evaluation metrics for error correction tasks encompass multiple aspects, often requiring considerations of trade-offs and compromises. As a result, providing a straightforward judgment regarding the superiority or inferiority of a specific method is frequently challenging. To visually illustrate the performance of each method on a unified chart, we have utilized Min–Max Normalization to standardize the metrics, excluding time and memory requirements. This normalization process aims to mitigate scale differences among different indicators. The formula for this normalization process is outlined as follows:

1 $${X}_{normalized}=\frac{X-{X}_{\text{min}}}{{X}_{\text{max}}-{X}_{\text{min}}}$$

Where, ${X}_{normalized}$ is the normalized value, $X$ is the original data value, ${X}_{\text{min}}$ is the minimum value of the original data, ${X}_{\text{max}}$ is the maximum value of the original data. In a normalized graph, when metrics derived from a specific algorithm are densely clustered near 1, it indicates that this algorithm may offer better overall performance compared to other algorithms whose metrics are more sparsely distributed and deviate significantly from 1, without major trade-offs or compromises.

### Analysis of the results

**Fig. 11:**
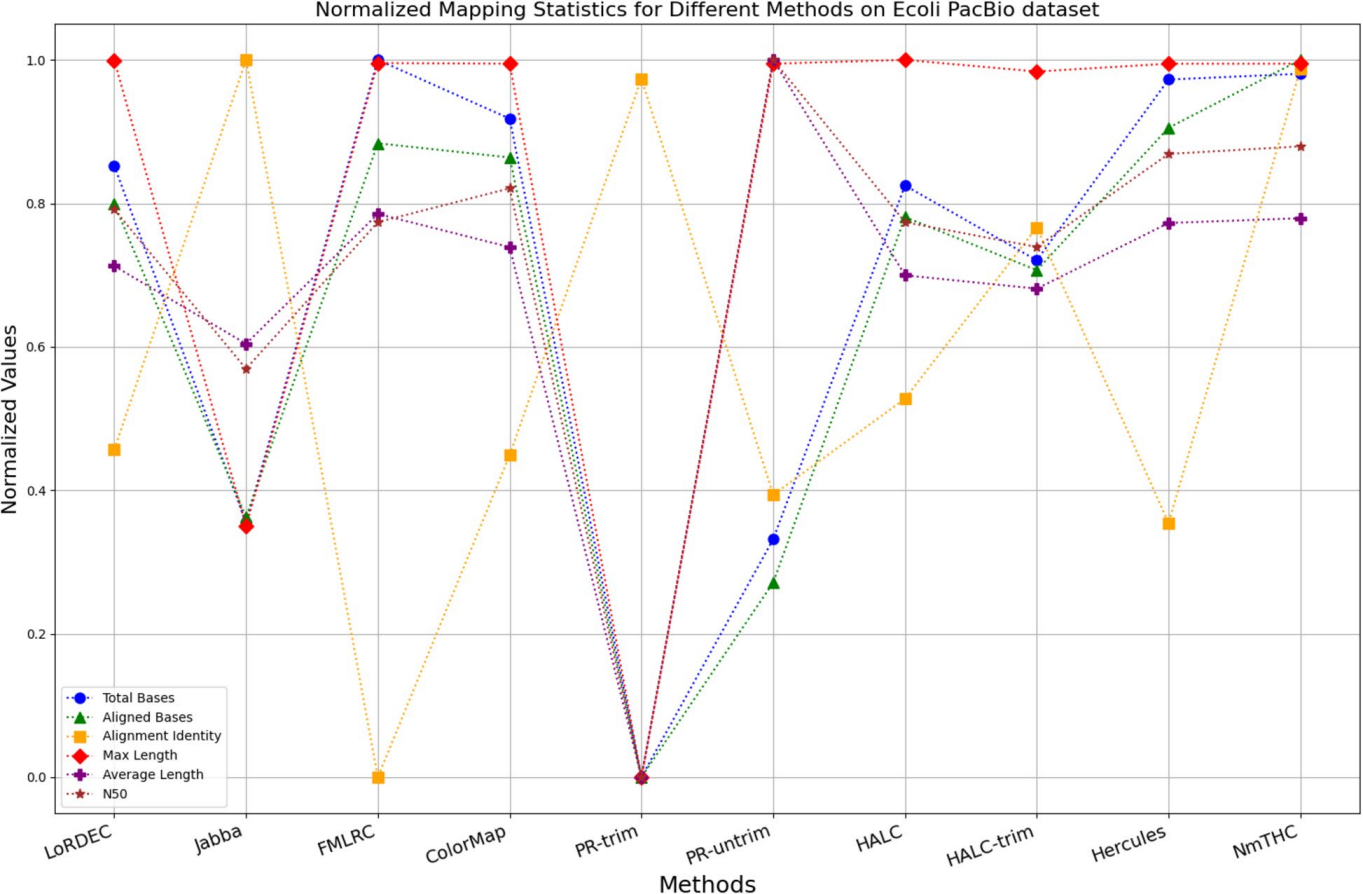
Legend of normalized metrics on E.coli PacBio dataset

**Fig. 12:**
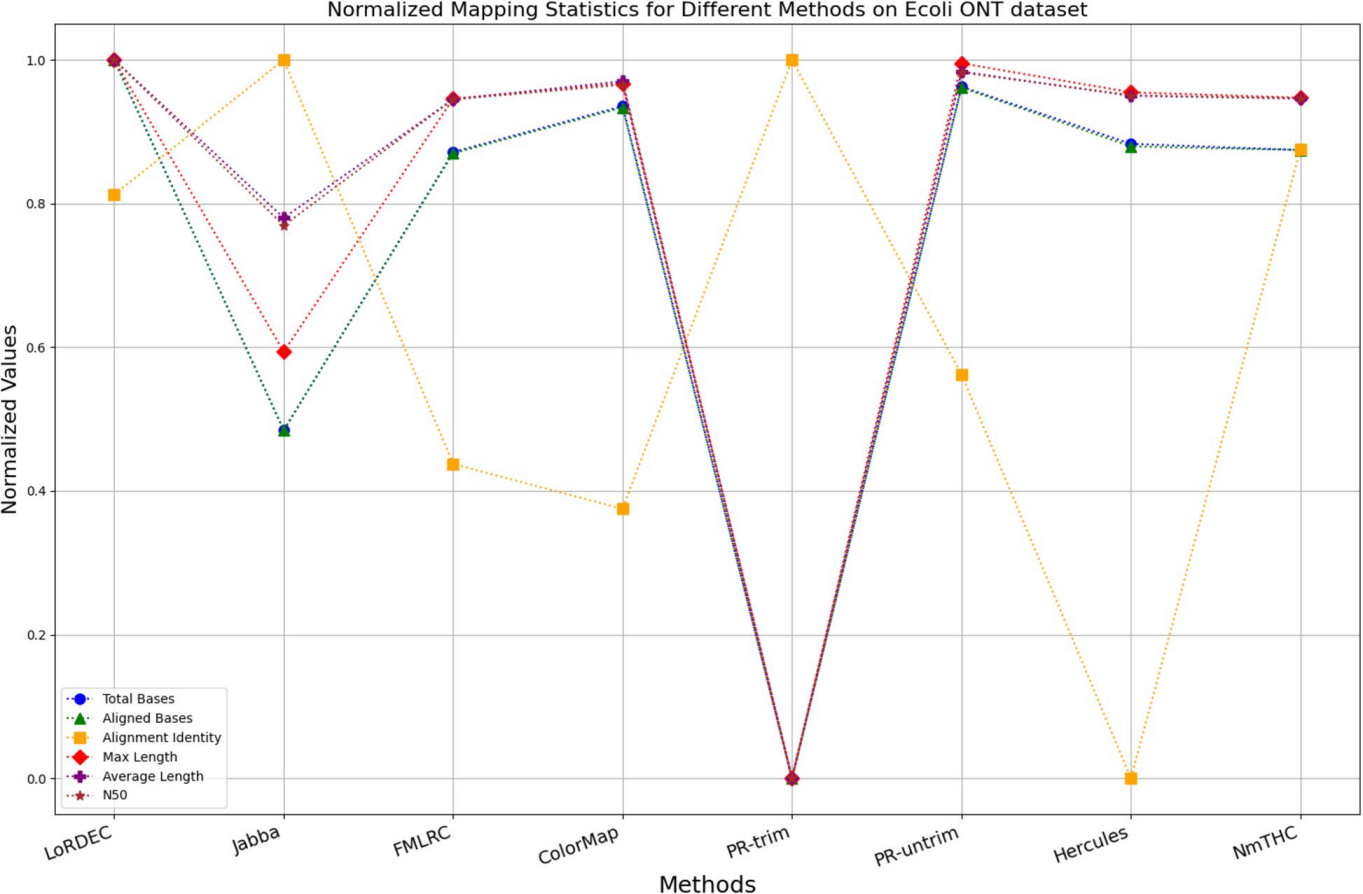
Legend of normalized metrics on E.coli ONT dataset

**Fig. 13:**
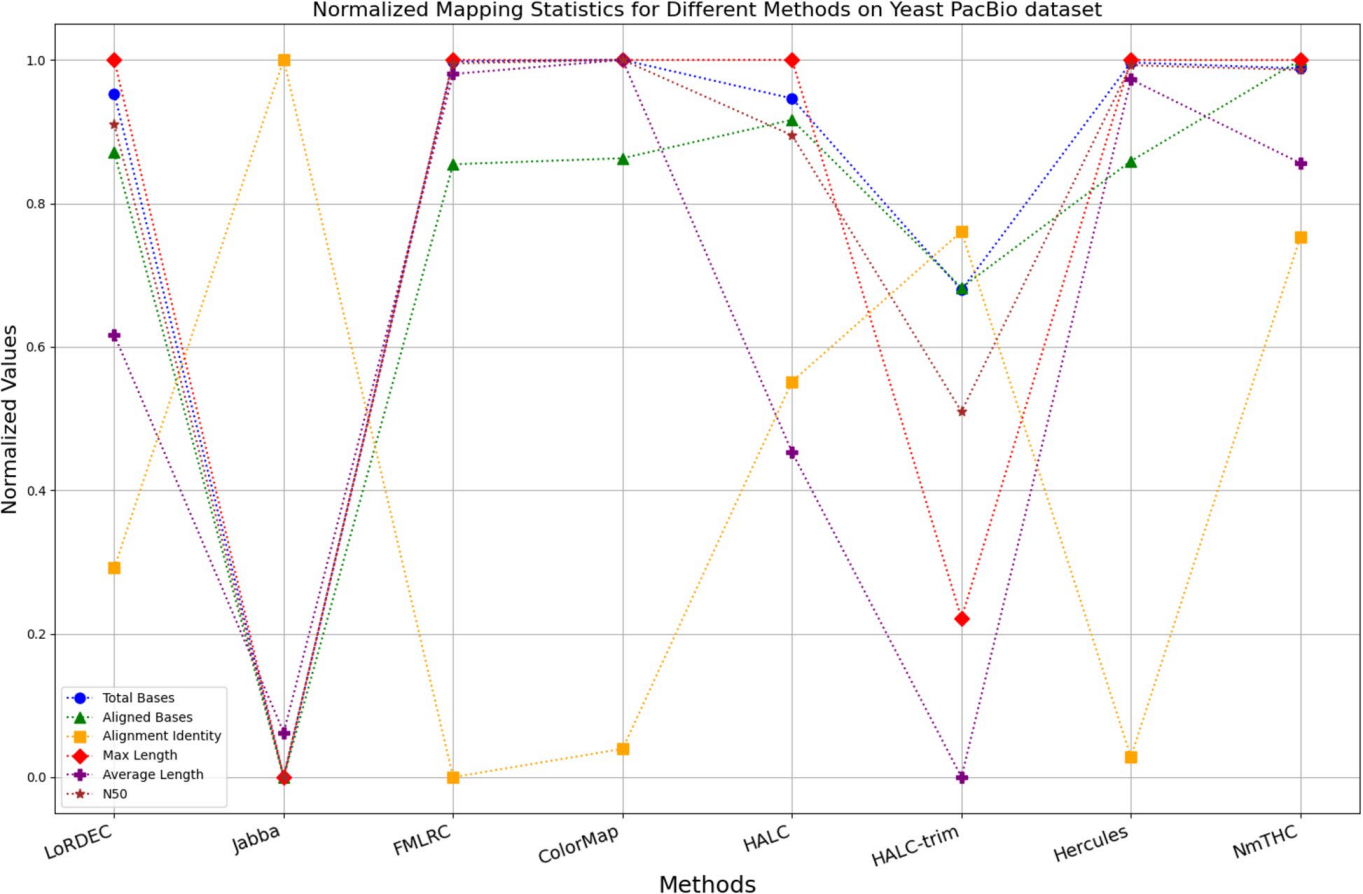
Legend of normalized metrics on Yeast PacBio dataset

**Fig. 14:**
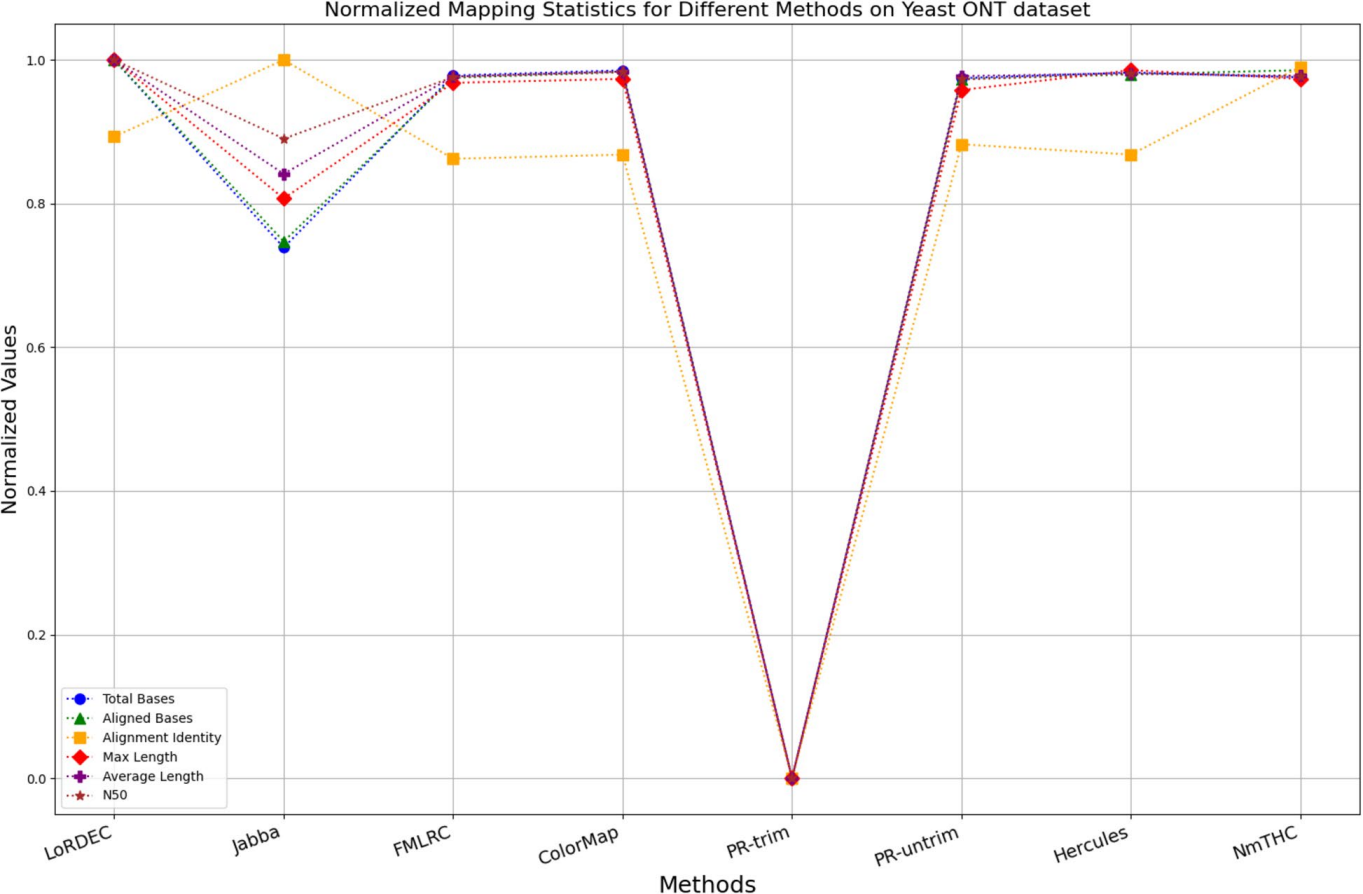
Legend of normalized metrics on Yeast ONT dataset

**Fig. 15:**
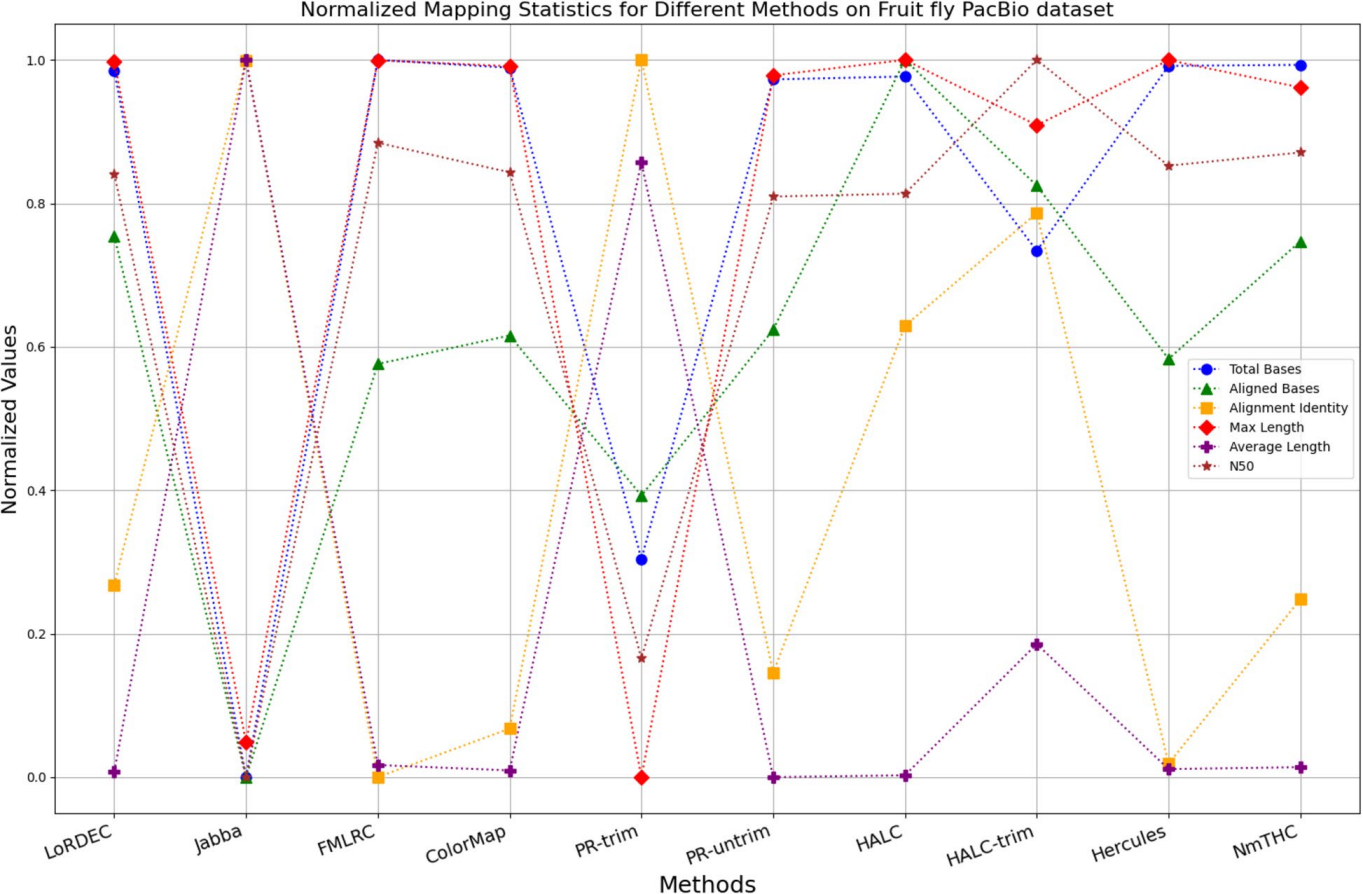
Legend of normalized metrics on Fruit fly PacBio dataset

**Fig. 16:**
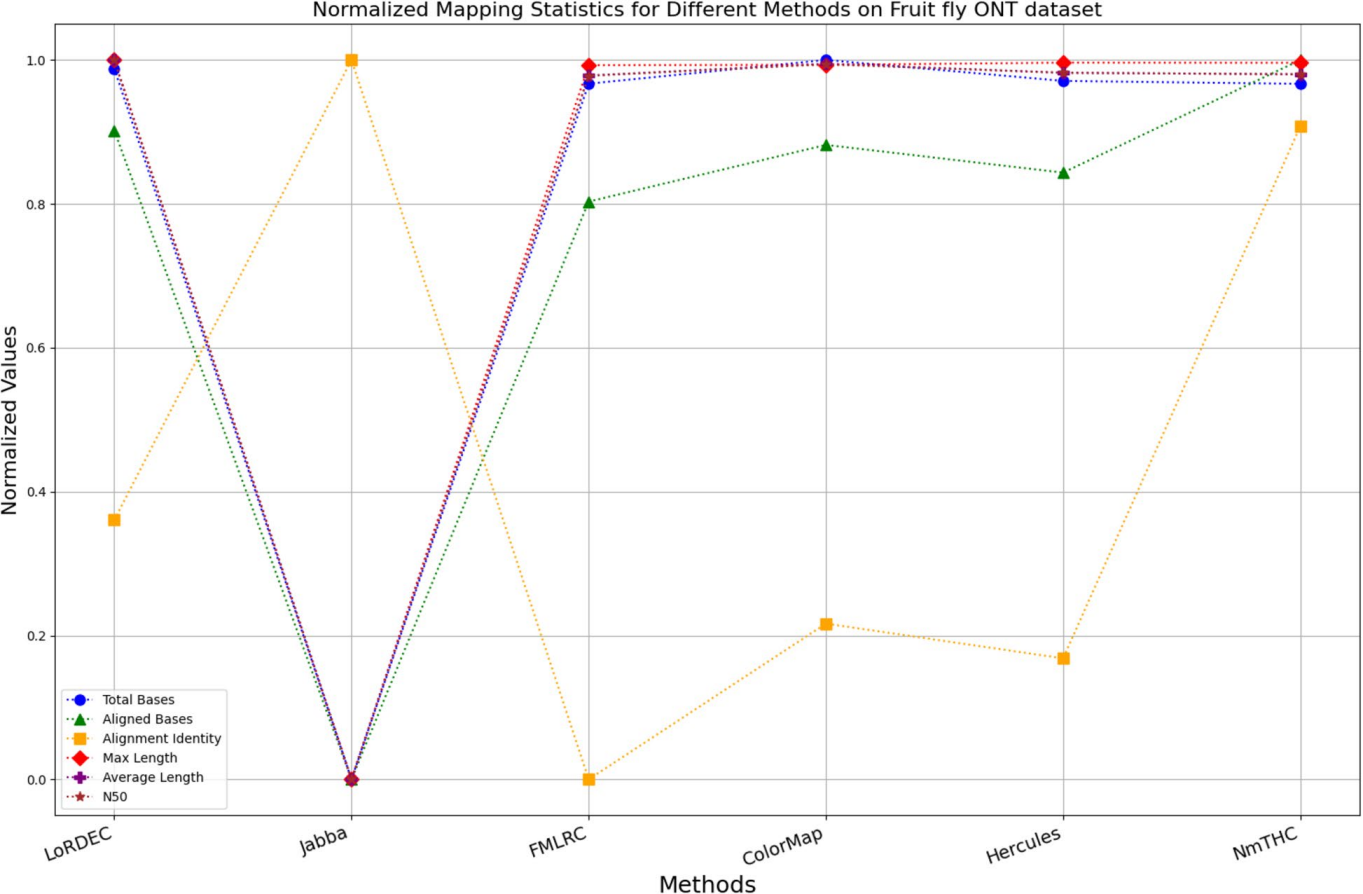
Legend of normalized metrics on Fruit fly ONT dataset

While enhancing the alignment identity of long reads with the reference genome is a crucial objective in error correction, it is imperative to consider a comprehensive set of metrics. Figures 11, 12, 13, 14, 15 and 16 depict the normalized metrics for each benchmark dataset. From the graph, it can be observed that for five of the six datasets, except for the fruit fly PacBio dataset, the indicators for NmTHC are more closely clustered around 1, indicating that it performs better with no significant weaknesses.

#### Trimming consideration

In Tables 3, 4, 5, 6, 7 and 8, Jabba appears to achieve an impressive alignment identity metric ranging from 0.99 to 1.00 in most cases, primarily due to its trimming strategy. Specifically, when long reads extend beyond the paths in the constructed De Bruijn graph, Jabba trims the extended extremities. While this trimming strategy elevates identity metrics, it results in significantly smaller values for metrics such as lengths and N50 compared to other algorithms. Consequently, this leads to the loss of global information and the length advantages inherent in long reads. Additionally, the long-read file corrected by Jabba is only one-third of its original size, a pattern similarly observed with Proovread. In Figs. 11, 12, 13, 14, 15 and 16, it is apparent that Jabba and Proovread occasionally show increased alignment identity. However, there are noticeable trade-offs in other metrics, such as total bases and the maximum read length. As a result, the markers representing these two methods in the figures display greater dispersion. When researchers are performing subsequent analyses, they should be careful of the utilization of both methods.

#### DBG-based algorithms

It is undeniable that the DBG-based algorithm LoRDEC is indeed an outstanding algorithm, and subsequent algorithms like HALC are derived from it. Admittedly, NmTHC is also quite comparable to it. Specifically, as shown in Tables 4 and 6, LoRDEC excels in metrics related to the number of aligned bases, alignment identity, as well as length indicators and N50. However, Tables 3, 5, 7 and 8 shows that NmTHC surpasses LoRDEC in these metrics. Overall, in 4 out of the 6 data sets, NmTHC outperforms LoRDEC in terms of performance. The difference in each indicator between these two methods is within 5%, indicating a comparable error correction performance. The same conclusions can also be demonstrated in Figs. 11, 13, 15, and 16. Notably, Tables 8 and 9 highlight NmTHC’s remarkable performance in terms of the number of aligned bases and alignment identity, suggesting its capability to handle complex structures in fruit fly data. Despite the complexity of the structure, alignment information is the key for generating feature vectors and labels. As for another DBG-based algorithm, FMLRC demonstrates exceptionally high error-correction throughput and saves computation time. However, based on results from six sets of data, its error-correction performance is somewhat disappointing.

**Table 9:** Parallel GPU time consumption test on E.coli PacBio dataset

Method	Aligned reads	Total bases	Aligned bases	Alignment identity	Average length (bp)	Maximum length (bp)	N50 (bp)	Real time (m:s)
NmTHC-1	85086	743904487	743534260	0.9995	8723	44113	13941	2606:49
NmTHC-2	85034	743583020	743212642	0.9995	8725	44113	13936	1464:51

“NmTHC-1” means there is 1 GPU working alone. “NmTHC-2” means there are 2 GPUs working in parallel

#### The only machine learning-based algorithm

The effectiveness of NmTHC is evident in Tables 3, 4, 5, 6, 7 and 8, where it enhances the count of aligned bases, improves alignment identity, and maintains a read length nearly equivalent to that of Hercules. This observation substantiates the assertions made in the introduction for the following reasons: 1) both methods report post-corrected long read sequences without trimming. 2) uncovered regions in short reads are effectively corrected leveraging the RNN’s capacity to capture long-term dependencies from the adjacent covered areas. 3) the approach is adaptable to diverse error profiles, making it suitable for various mainstream sequencing platforms. In conclusion, NmTHC surpasses the state-of-the-art machine learning-based method Hercules across all metrics while significantly reducing user time requirements.

#### Preassembly-based algorithm

For the fruit fly dataset obtained from the PacBio platform, as illustrated in Table 7 and Fig. 15, a noticeable dissimilarity exists between the original long-read data and the reference genome, leading to a low alignment identity. In the results of Jabba and Proovread, there is a peculiar doubling of the average length accompanied by a significant reduction in total bases and maximum length. This is because these two methods exclude many long-read fragments that cannot be aligned to the high-precision short reads or the DBG constructed from these short reads.

For such sequencing data with significant noise, HALC offers a solution by initially using a third-party assembler, Abyss [38], to preassemble the short-read data to contigs. During this preassembly phase, contaminants are removed with the ‘–chastity’ option, low-quality bases at the end of sequences are trimmed with ‘–trim-masked’, and dangling edges are pruned with the ‘-t’ parameter. The obtained clean contigs and short reads are then used to construct a DBG, followed by further refinement of the long reads with LoRDEC. In the case of the fruit fly PacBio dataset, this strategy has proven to be quite effective. However, the complex assembly process consumes considerable time and computational resources, and the preassembly approach is not suitable for other tested datasets. In addition, HALC is designed only for SMRT PacBio long reads.

Unlike the previous trimming or pre-assembly strategies, the other algorithms retained these regions, which results in a suboptimal alignment identity. Nevertheless, NmTHC still reports higher alignment identity and a greater number of aligned bases on this dataset.

### Resource consumption statistics

In terms of computing resource consumption, the tokenization process of “long sentences” and “target sentences” and the generation of vocabularies of NmTHC method are executed on the CPU, and a total of 3.0GiB of main memory is costed. Then, the training and prediction of the model are deployed onto the GPU, and a total of 23181MiB of GPU memory is consumed. The data generator is used to load “long sentences” in batches for training and prediction, only the current batch of data and the parameters of the model are saved in the GPU. Thus, the GPU memory consumed is fixed regardless of the size of long reads such as fruit fly and the only concern is the running time. Other compared algorithms only consume main memory, and their memory consumption is shown in the “Memory usage” column in the above tables.

In terms of running time, it is well established that generative models are time-consuming and computationally resource-intensive. To evaluate the time efficiency of the algorithms, the Unix “time” command is used to record the running time of each method, then the command line will output three values, “real time” is the elapsed real (wall clock) time used by the process, “user time” is the CPU-seconds used by process directly in user mode. It is worth noting that when multiple cores in the CPU are called, the “real time” may be smaller than “user time”. The number of CPU cores specified by each method is different, “user time” is used here to measure the time consumption. The machine learning-based method Hercules is obviously time-consuming.

In recent years, there has been rapid development in hardware GPUs and the cloud GPU arrays. As a machine learning-based hybrid error correction method running on GPUs, NmtHC can further enhance its time efficiency with the advancement of GPU resources. To validate this, the training process is distributed in parallel across two GPUs with the interface “*tensorflow.config*” in Keras library. It is necessary to double the “batch_size” and specify that no regularization is performed between GPUs. The results are shown in Table 9. The process of alignment takes 170 min of user time. Thus, the actual user time spent on 2 GPUs is almost half that of 1 GPU. The corrected long-read quality obtained from a model trained by parallel GPUs is comparable to that of a single GPU-trained model. This shows that if there are sufficient GPUs or cloud GPU arrays, our algorithm can achieve faster high-precision error correction.

## Conclusions

This work employs the idea of NLP to realize an NMT-based Hybrid Correction (NmTHC) method which adopts a RNN to build a seq2seq framework, treating the long reads to be corrected as the sentences in the source language and the corrected long reads as the sentences in the target language, realizing the error correction with the help of the special corpus generated from the alignment information between long and the high precision short reads.

The proposed method can automatically capture longer-term dependencies among sequences and identify the discrepancies between long and short reads for error correction. This benefits an improvement in alignment identity while maintaining a high count of total bases and aligned bases. NmTHC avoids trimming any uncovered bases and leverages long-term correlation to correct them. As a result, NmTHC performs better than other mainstream error correction algorithms, including efficient LoRDEC and autonomous learning Hercules. NmTHC offers a fresh perspective for machine learning-based error correction tasks.

## Data Availability

The source code for NmTHC is available at https://github.com/Beauty9527/NmTHC.
